# The relationship between white matter hyperintensity and gait in patients with cerebral small vessel disease

**DOI:** 10.3389/fneur.2026.1909850

**Published:** 2026-08-25

**Authors:** Yajing Wang, Zhizhong Zhu, Jiaojiao Zhao, Shoufeng Liu

**Affiliations:** 1 Clinical College of Neurology, Neurosurgery and Neurorehabilitation, Huanhu Hospital Affiliated to Tianjin Medical University, Tianjin, China; 2 Department of Neurology, Tianjin Huanhu Hospital, Tianjin Key Laboratory of Cerebral Vascular and Neurodegenerative Diseases, Tianjin Neurosurgical Institute, Tianjin, China; 3 Department of Rehabilitation, Tianjin Huanhu Hospital, Tianjin, China

**Keywords:** cerebral small vessel disease, gait analysis, magnetic resonance imaging, wearable device, white matter hyperintensities

## Abstract

**Background:**

Recent studies have explored the link between white matter hyperintensity (WMH) and gait disorders; however, inconsistent results have arisen due to varied populations and sample sizes. We aimed to examine the relationship between WMH severity and gait in patients with cerebral small vessel disease (CSVD).

**Methods:**

We included 335 patients with CSVD with independent walking ability hospitalized at the Department of Neurology, Tianjin Huanhu Hospital, from August 1, 2018, to May 17, 2021. WMH was graded using the Fazekas scale on magnetic resonance imaging. Patients were categorized into the mild and moderate-to-severe WMH groups. A wearable gait analysis system was employed to evaluate the impact of WMH severity on patients’ walking ability.

**Results:**

Patients in the moderate-to-severe WMH group exhibited slower gait speed, shorter length stride, and reduced foot strike and toe-off angles compared to those in the mild WMH group, all with significant differences (*p* < 0.05). Analysis of gait parameter symmetry and variability showed significant increases in the moderate-to-severe WMH group. Multivariate logistic regression analysis identified toe-off angle, swing phase coefficient of variation, and age as independent risk factors for WMH-induced gait impairment.

**Conclusion:**

The severity of white matter lesions affects the gait of patients with small vessel disease, including spatiotemporal and kinematic parameters, symmetry, and variability. With increasing age, the severity of white matter lesions increases, resulting in a reduced toe-off angle and greater swing variability, leading to decreased stability.

## 1 Introduction

Cerebral small vessel disease (CSVD) is a syndrome characterized by clinical, imaging, and pathological signs stemming from various structural or functional lesions in small intracranial arteries, penetrating arteries, capillaries, and small veins (1). White matter hyperintensity (WMH) of presumed vascular origin (WMH) is one of the main neuroimaging markers of CSVD (2). The primary manifestation of WMH is symmetrical hyperintensity on T2-weighted magnetic resonance imaging (MRI) of both cerebral hemispheres, which may also extend into the surrounding brain tissue that appears normal (3). WMH is prevalent among older adults in the community and is a leading cause of gait disorders in CSVD imaging biomarkers, increasing the risk of falls and disabilities (4, 5). The Leukoaraiosis and Disability Study (LADIS) revealed a strong correlation between severe deep frontal lobe WMH burden and gait, specifically manifesting as gait impairments, balance disorders, and a higher incidence of slow walking speed (6). Moderate-to-severe WMH was found to be independently associated with declining gait and balance function. In recent years, studies on the relationship between WMH and gait disorders have yielded inconsistent results owing to differences in study populations and sample sizes. These investigations have primarily focused on conventional spatiotemporal parameters, such as gait speed, step length, and cadence, leaving a notable gap in the detailed analysis of more subtle yet clinically critical kinematic variables (4, 7, 8). In particular, the toe-off angle—a parameter increasingly recognized as a sensitive indicator of distal motor control and closely linked to fall risk in CSVD populations—has received limited attention in the context of WMH grading using wearable systems. Therefore, building upon the established findings of previous studies, the present study employed the Fazekas scale for MRI-based WMH grading in conjunction with a wearable gait analysis system. We aimed to specifically investigate how different grades of WMH affect the toe-off angle and other detailed gait parameters, with the goal of establishing earlier and more precise identification criteria for gait impairment caused by WMH.

## 2 Methods

### 2.1 Subjects

This study included 335 patients with CSVD who met the inclusion criteria and were admitted to the Neurology Department of Tianjin Huanhu Hospital between August 17, 2018, and May 17, 2021. Prior to participation, all subjects and their families received detailed information about the investigation processes and signed an informed consent form. This study received approval from the Ethics Committee of Tianjin Huanhu Hospital and was registered with the Chinese Clinical Trial Registry (Clinical Trial Registration No. ChiCTR2100042031). Each participant underwent a head MRI and demonstrated independent walking ability. All patients were capable of completing assessments without assistance, and their clinical data were complete.

The inclusion criteria were: (1) meeting the STandards for ReportIng Vascular changes on nEuroimaging (STRIVE) (9) attesting to the criteria for CSVD and WMH diagnosis; (2) ability to walk independently and safely for 2 min without aid or assistive devices; and (3) no use of medication that could affect functional assessment.

Exclusion criteria comprised: inability to undergo head MRI examination, clinical diagnosis of dementia, mental disorders, severe cerebral hemorrhage, and other systemic diseases impacting gait such as joint injuries, arthritis, cervical spondylosis, lumbar spondylosis, and osteoporosis.

### 2.2 Data collection

We collected the general clinical data of all patients, including age, sex, height, weight, hypertension, diabetes, cardiac disease, and smoking history.

#### 2.2.1 MRI examination

Head MRI examinations were conducted on all patients using a 3.0 T MRI scanner while in a resting state. This included T1-weighted imaging (T1WI), T2-weighted imaging (T2WI), T2 fluid-attenuated inversion recovery (FLAIR) sequences, diffusion-weighted imaging (DWI) sequences, and gradient echo (GRE) sequences.

#### 2.2.2 MRI evaluation method

Based on the Fazekas scale (10), WMH was classified into deep WMH (DWMH) and periventricular WMH (PVWMH) categories and scored separately. The DWMH score was “0” for no white matter hyperintensities and “l” for speckled hyperintensities. A score of “2” indicated patchy high signal (with a tendency for fusion between lesions) or single lesion diameter >3 mm, while a score of “3” indicated patchy and irregular high signals (lesions were extensively fused). PVWMH was scored as “0” for no white matter hyperintensities, “1” for hat-shaped or pencil-shaped thin layer hyperintensities in the frontal or occipital horn of the lateral ventricle, “2” for smooth and circular hyperintensities in the lateral ventricle, and “3” for irregular PVWMH penetrating deep into the white matter. Based on clinically established thresholds distinguishing none-to-minimal lesions from confluent or deeply extending lesions (11), subjects with a DWMH score of 0–1 or PVWMH score of 0–2 were classified into the mild WMH group, as these scores represent lesions without confluence or deep extension. Subjects with a DWMH score ≥2 or PVWMH score ≥3 were classified into the moderate-to-severe WMH group, as these scores indicate confluent or deeply extending lesions that have been consistently associated with significant white matter pathology and adverse clinical outcomes (12).

#### 2.2.3 Gait parameter measurement

**Figure 1:**
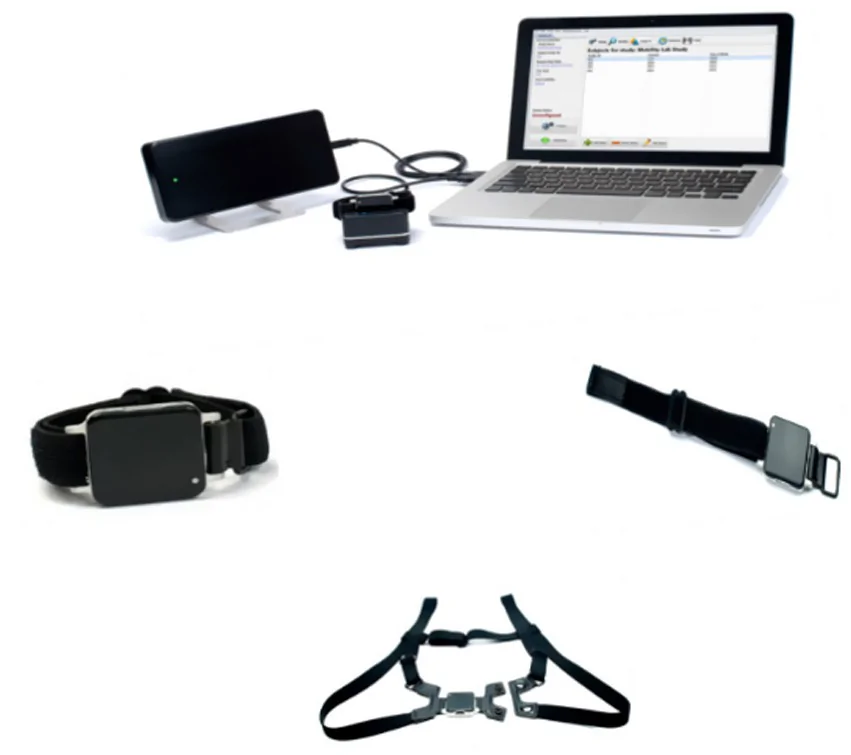
Hardware composition of the wearable inertial gait analysis system. This figure displays the complete hardware setup of the gait detection system. The upper part shows the data receiving base station connected to a laptop with supporting analysis software interface; The lower part presents wearable inertial sensor modules matched with wrist straps, shank straps and torso harness, which are fixed on corresponding body segments to capture three-dimensional kinematic gait signals during walking trials.

This study utilized a wearable gait analyzer (OPAL wearable sensor, APDM Company, Portland, Figure 1) to assess the gait characteristics of the study subjects during walking and to obtain relevant parameters. At the start of the experiment, the subjects wore six inertial sensors, each of which was securely fastened to the following body parts by professionals using nylon buckles: the first sensor was positioned at the midline of the sternum; the second sensor was approximately located at the fifth lumbar vertebra; the third and fourth sensors were placed at the proximal ends of both wrist joints; and the fifth and sixth sensors were affixed to the back of both feet. Several studies have reported the calculation method, accuracy, and effectiveness of this device (13, 14).

All enrolled patients performed the 7-m walking test under the guidance of a physician (15). Prior to formal data collection, all participants underwent two practice runs to familiarize themselves fully with the start signal, turning position, and termination instructions. Although the participants followed the same routine during practices and formal tests, we neither recorded nor analyzed the practice data. Before the commencement of the formal test, participants were instructed to stand behind the starting line. The inertial motion sensor system emitted the first verbal prompts, following which the participants were required to look straight ahead at a fixed object and remain stationary in a natural standing posture for 30 s. Upon hearing the second prompt, participants began to walk forward at a natural and comfortable pace. After crossing the ground-marked line (the 7-m blue line), participants turned around and walked back toward the starting line. Upon crossing the starting line, the system verbally prompted “Stop,” and participants were instructed to stand still immediately, marking the end of the test. Data acquisition ceased simultaneously. Only the data from the first formal test were subsequently analyzed. If the formal test was interrupted (e.g., by a sudden noise or personnel passing through the testing area), the test was invalidated and then repeated after the participant had been given adequate rest. A rest period of at least 1 min in a sitting position was arranged between each formal test to avoid the possibility that fatigue may affect the gait performance.

#### 2.2.4 Gait parameter data collection

**Figure 2:**
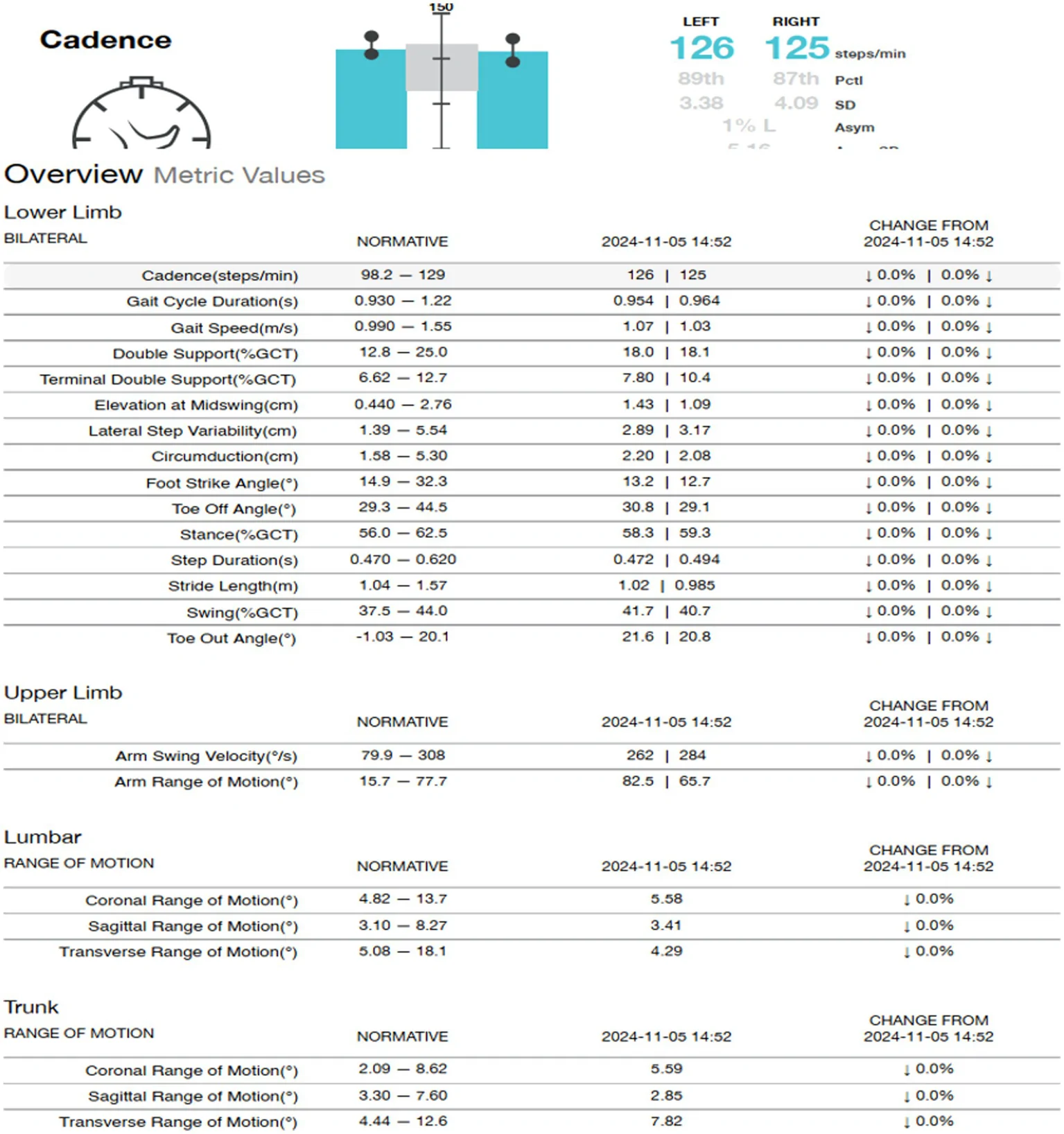
Overview interface of quantitative gait metric output from wearable inertial gait analysis software. The top bar chart displays cadence data of left and right lower limbs; the main table systematically summarizes bilateral gait parameters categorized into Lower Limb, Upper Limb, Lumbar and Trunk modules. Each row includes normative reference ranges, subjects’ measured values, and percentage changes compared with the previous test record, covering spatiotemporal gait indicators and multi-plane joint range of motion metrics.

Gait parameter data were collected using a gait analysis testing system and then transmitted to a computer terminal for further analysis of the spatiotemporal and kinematic parameters (Figure 2). The calculation and analysis of the gait parameter symmetry was based on the asymmetry index (AI) (16), which was calculated as follows:

AI = |XL-XR|/ max(XL, XR) × 100.

Here, *L* and *R* denote the patient’s left and right sides, respectively, and *X* was the corresponding gait parameter. The analysis and evaluation of the variability of the gait parameters were based on the coefficient of variation (CV) (17). Initially, the CV, which corresponded to the variability of the, gait was calculated as.

“standard deviation (SD)/mean” (i.e., CVL: left variability; CVR: right variability). The gait parameter variability on both sides was then determined as “ $\sqrt{(\mathrm{CVL}+\mathrm{CVR})/2\times 100.}$ ”

**Figure 3:**
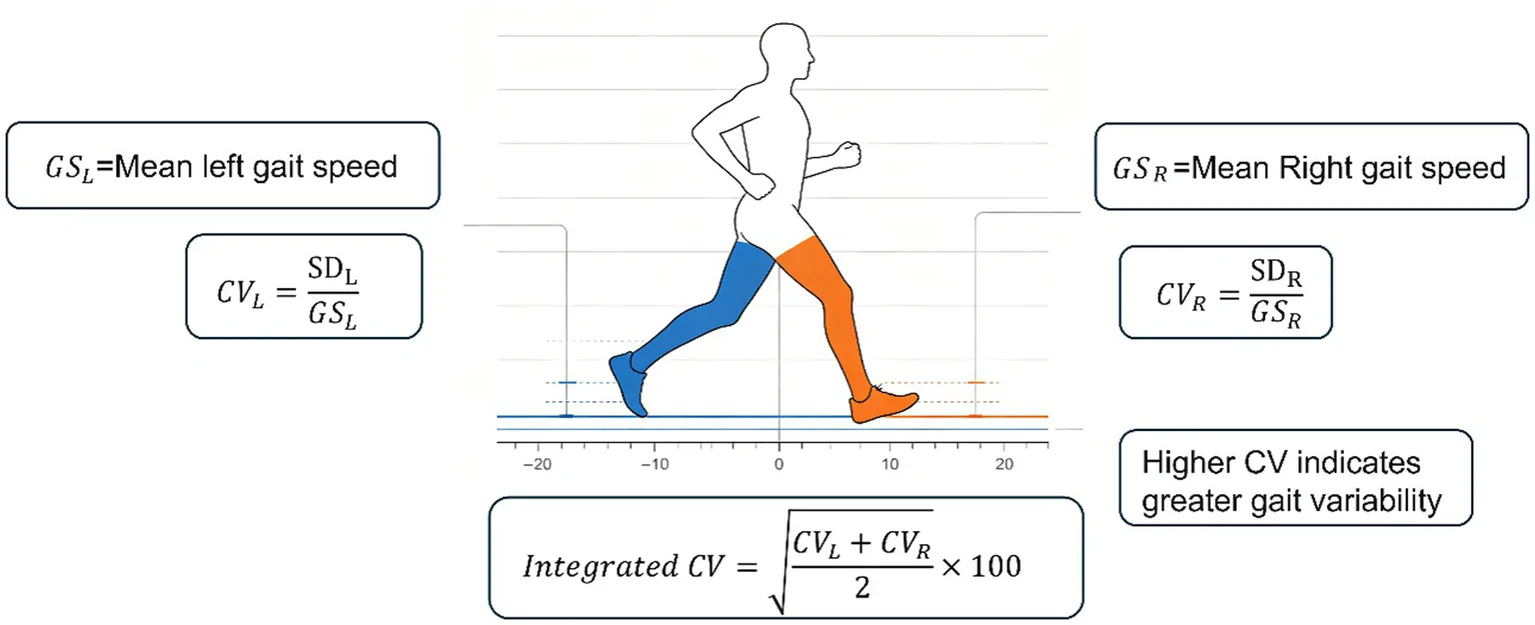
An example of gait speed variability. SD is the standard deviation and mean is the average of gait speed across all gait cycles. A higher coefficient of variation (CV) indicates greater stride-to-stride variability, indicating poorer gait control stability.

Regarding the gait speed variability, SD refers to the standard deviation of the gait speed across all gait cycles, whereas the mean is the average value of gait speed across all gait cycles. A higher CV indicates greater stride-to-stride variability in gait speed, reflecting poorer stability of gait control (Figure 3).

#### 2.2.5 Statistical analysis

SPSS Statistics for Windows, version 24.0 (IBM Corp., Armonk, NY, United States) was used for data analysis. Quantitative data with a normal distribution are presented as mean ± SD ( $\bar{x}$ ± s), and an independent sample t-test was used for inter-group comparison. Quantitative data with skewed distribution are presented as median (first quartile, third quartile) [*M* (Q1, Q3)], and inter-group comparisons are performed using Mann–Whitney *U* test. Categorical data are expressed as frequency (percentage), and the chi-square test was used for inter-group comparison. A significance level of *p* < 0.05 was considered statistically significant. Logistic regression analysis was performed to determine significant associated factors gait data, with *p* < 0.05 indicating statistical significance.

## 3 Results

### 3.1 Participant clinical characteristics

This study comprised 335 patients with CSVD (mean age, 60.07 ± 9.86 years; 254 males and 81 females) who were categorized into a mild WMH group (*n* = 217) and a moderate-to-severe WMH group (*n* = 118). The mild WMH group exhibited significantly lower average age and incidence of hypertension and cardiac disease (*p* < 0.05). No significant differences were observed in sex, height, weight, prevalence of diabetes, or smoking between the two groups (*p* > 0.05; Table 1).

**Table 1:** Comparison of general clinical data between the two groups (mild WMH and moderate-to-severe WMH).

Gait Parameters	Mild WMH group (*n* = 217)	Moderate-to-severe WMH group (*n* = 118)	*X*^2^/*t* value	*p*-value
Male [*n* (%)]	164(75.6)	90(76.3)	0.020	0.887
Age (year)	58.07 (year)t	63.74 (year)t	−5.187	<0.001
Height (cm)	168.79 (cm)o	169.35(cm)o	−0.627	0.531
Weight (kg)	58.07 t(kg)	63.74 t(kg)	−0.677	0.499
Hypertension [*n* (%)]	127(60.5)	88(78.6)	10.780	0.001
Diabetes [*n* (%)]	68(32.2)	33(29.2)	0.314	0.575
Cardiac Disease [*n* (%)]	27(12.9)	25(22.3)	4.832	0.028
Smoke [*n* (%)]	123(58.3)	62(54.9)	0.353	0.553

WMH, white matter hypersensitivity.

### 3.2 Comparison of gait parameters between WMH groups

Patients in the moderate-to-severe WMH group displayed slower gait speed and shorter stride length compared to those in the mild WMH group, with statistically significant differences (*p* < 0.05). Additionally, patients in the moderate-to-severe WMH group exhibited significantly reduced foot strike angle and toe-off angle compared to those in the mild WMH group (both *p* < 0.001). However, no significant differences were noted in the cadence, gait cycle duration, double support, single limb support, terminal double support, swing, lumbar-coronal range of motion, lumbar-sagittal range of motion, lumbar-transverse range of motion, upper limb-arm swing velocity, and arm range of motion (*p* > 0.05; Table 2).

**Table 2:** Comparison of gait parameters between the two groups ( $\bar{x}$ ±s).

Gait Parameters	Mild WMH group (*n* = 217)	Moderate-to-severe WMH group (*n* = 118)	*t* value	*p*-value
Cadence (steps/min)	105.76 ± 11.13	103.58 ± 12.23	1.649	0.100
Gait speed (m/s)	0.92 ± 0.23	0.83 ± 0.24	3.232	0.001
Stride length (m)	1.02 ± 0.19	0.94 ± 0.22	3.550	<0.001
Gait cycle duration (s)	1.15 ± 0.13	1.18 ± 0.16	−1.779	0.076
Double support (%GCT)	23.15 ± 5.83	24.41 ± 6.62	−1.806	0.072
Single limb support (%GCT)	38.40 ± 2.89	37.78 ± 3.32	1.770	0.078
Terminal double support (%GCT)	11.64 ± 2.87	12.27 ± 3.29	−1.823	0.069
Swing (%GCT)	38.42 ± 2.93	37.78 ± 3.30	1.839	0.067
Foot strike angle (°)	18.33 ± 6.01	15.73 ± 6.68	3.632	<0.001
Toe off angle (°)	32.50 ± 5.23	29.61 ± 6.17	4.524	<0.001
Lumbar-coronal range of Motion (°)	4.87 ± 2.02	4.83 ± 1.60	0.169	0.866
Lumbar-Sagittal Range of motion (°)	5.10 ± 1.82	5.12 ± 1.86	−0.057	0.954
Lumbar-transverse range of motion (°)	8.13 ± 2.93	8.25 ± 2.87	−0.363	0.717
Upper limb-arm swing velocity (°/s)	150.90 ± 60.33	140.67 ± 51.40	1.560	0.120
Arm range of motion (°)	34.41 ± 16.64	32.38 ± 14.60	1.117	0.265

WMH, white matter hypersensitivity.

### 3.3 Comparison of gait parameter symmetry

The AI of the foot strike angle in patients with the moderate-to-severe WMH group increased significantly compared to those in the mild WMH group (*p* < 0.05). However, no significant difference was noted in the cadence AI, gait speed AI, stride length AI, single limb support AI, swing AI, and toe-off angle AI (*p* > 0.05; Table 3).

**Table 3:** Comparison of gait symmetry between the two groups [%, *M*(Q1, Q3)].

Gait Parameters	Mild WMH group (*n* = 217)	Moderate-to-severe WMH group (*n* = 118)	*Z* value	*p*-value
Cadence AI	0.57(0.26, 0.94)	0.51(0.24, 1.08)	−0.003	0.998
Gait speed AI	2.20(0.99, 5.09)	2.44(1.31, 4.91)	−1.062	0.288
Stride length AI	2.29(1.01, 4.83)	2.88(1.56, 4.43)	−1.708	0.088
Single limb Support AI	2.85(1.48, 5.84)	2.58(1.46, 5.87)	−0.351	0.726
Swing AI	3.27(1.21, 6.07)	2.97(1.51, 5.96)	−0.276	0.782
Foot strike angle AI	17.50(8.80, 30.01)	18.91(10.83, 32.55)	−2.205	0.027
Toe-off angle AI	6.38(3.38, 11.64)	5.77(3.88, 11.46)	−0.848	0.396

WMH, white matter hypersensitivity; AI: asymmetry index.

### 3.4 Comparison of gait parameter variability

Patients in the moderate-to-severe WMH group exhibited significantly higher gait speed CV, stride length CV, double support CV, single limb support CV, swing phase CV, foot strike angle CV, and toe-off angle CV compared to those in the mild WMH group (*p* < 0.05; Table 4).

**Table 4:** Comparison of gait parameter variability between the two groups [%, *M*(Q1, Q3)].

Gait Parameters	Mild WMH group (*n* = 217)	Moderate-to-severe WMH group (*n* = 118)	*Z* value	*p*-value
Cadence CV	19.73(17.32, 22.37)	20.97(17.77, 24.34)	−1.661	0.097
Gait speed CV	29.38(25.90, 34.68)	31.67(26.87, 38.06)	−1.996	0.046
Stride length CV	25.61(21.27, 30.74)	27.61(23.68, 35.78)	−2.655	0.008
Gait cycle duration CV	19.84(17.42, 22.56)	21.12(18.39, 24.50)	−1.641	0.101
Double support CV	28.48(24.75, 32.45)	29.93(26.35, 35.42)	−2.197	0.028
Single limb Support CV	18.04(15.32, 21.37)	19.20(16.86, 23.30)	−2.942	0.003
Terminal double Support CV	32.39(28.72, 37.01)	33.90(30.14, 38.38)	−2.428	0.015
swing phase CV	17.99(15.92, 23.65)	19.79(17.29, 24.22)	−3.124	0.002
Foot strike angle CV	40.71(33.35, 50.31)	44.62(35.92, 55.22)	−3.132	0.002
Toe-off angle CV	26.48(21.50, 30.95)	28.73(23.95, 33.47)	−2.001	0.045

WMH, white matter hypersensitivity; CV, coefficient of variation.

### 3.5 Multivariate regression analysis

All statistically significant gait indicators were included in the multivariable logistic regression analysis. Significant differences (*p* < 0.05) occurred between the two groups in toe-off angle, swing phase CV, and age, which were independent risk factors affecting walking ability in patients with WMH. However, the other spatiotemporal, kinematic, symmetry, and variability parameters did not show statistical significance (*p* > 0.05; Table 5).

**Table 5:** Results of the multivariable logistic regression analysis.

Gait Parameters	β result	SE	Wald value	*p*-value	OR value	95% CI	
						Lower limit	Upper limit
Gait speed	−0.111	0.261	0.181	0.671	0.895	0.536	1.494
Stride length	0.239	0.352	0.461	0.497	1.269	0.637	2.528
Foot strike angle	0.125	0.071	3.140	0.076	1.133	0.987	1.301
Toe-off angle	−0.166	0.076	4.754	0.029	0.847	0.729	0.983
Foot strike angle AI	0.012	0.013	0.909	0.340	1.012	0.987	1.039
Gait speed CV	−0.090	0.071	1.627	0.202	0.914	0.796	1.050
Stride length CV	0.122	0.078	2.469	0.116	1.130	0.970	1.315
Foot strike angle CV	0.037	0.030	1.519	0.218	1.038	0.978	1.101
Toe-off angle CV	0.010	0.054	0.032	0.859	1.010	0.909	1.122
Double support CV	0.051	0.084	0.367	0.545	1.052	0.893	1.240
Swing phase CV	−0.196	0.095	4.272	0.039	0.822	0.682	0.990
Double support CV	0.138	0.082	2.866	0.090	1.148	0.978	1.347
Terminal double support CV	−0.089	0.075	1.410	0.235	0.915	0.791	1.059
Age	0.062	0.024	6.970	0.008	1.064	1.016	1.115
Hypertension	0.330	0.377	0.767	0.381	1.391	0.664	2.915
Cardiac disease	0.363	0.512	0.504	0.478	1.438	0.527	3.921

CI, confidence interval; SE, standard error; OR, odds ratio; AI, asymmetry index; CV, coefficient of variation.

## 4 Discussion

### 4.1 Baseline characteristics and sex distribution

Patients in the moderate-to-severe WMH group were older and had a higher prevalence of hypertension and cardiac disease compared to those in the mild WMH group. Consistent with our findings, previous studies demonstrated that age and hypertension are independent risk factors for WMH in patients aged >60 years (18), and age correlated positively with increased incidence of WMH (19). Furthermore, we noted a significantly higher prevalence of cardiac disease in the moderate-to-severe WMH group than in the mild WMH group (*p* < 0.05). This could be closely linked to the long-term effects of elevated systolic and diastolic blood pressure on the cardiovascular and cerebrovascular systems. The male predominance in our cohort (254/335) may reflect both the higher burden of vascular risk factors in men leading to increased hospitalization for CSVD, and the disproportionate exclusion of women due to a higher prevalence of gait-limiting comorbidities (e.g., osteoarthritis, osteoporosis). This imbalance is consistent with previous reports stating that male patients with CSVD exhibit a heavier vascular risk profile and earlier disease onset (20).

### 4.2 Spatiotemporal and kinematic gait parameters

Patients with moderate-to-severe WMH exhibited significantly reduced gait speed, cadence, and step length compared with those in the mild WMH group, suggesting that greater WMH burden is associated with a generalized decline in motor function and physical fitness. This spatiotemporal pattern—slower walking speed accompanied by shorter steps—is consistent with previous reports (21, 22) and likely reflects a compensatory gait strategy adopted to maintain postural stability. This could possibly have been driven by reduced lower limb muscle strength, impaired central motor control, and deficits in the planning and execution of locomotor tasks.

Beyond these spatiotemporal alterations, we further observed significant reductions in both foot strike angle and toe-off angle in the moderate-to-severe WMH group. These kinematic changes collectively impair the ability to clear the ground effectively, thereby increasing gait instability and fall risk. Anatomically, WMH predominantly affects frontal lobe white matter tracts, including the anterior thalamic radiation and corticospinal pathways, impairing gait planning and distal lower limb muscle control (23, 24). The corticospinal and corticoreticular pathways, critical for ankle plantarflexors and dorsiflexors, are vulnerable to WMH related damage, possibly explaining the observed reductions in foot strike and toe-off angles. Notably, toe-off angle was as an independent predictor of WMH severity, underscoring its clinical relevance. Although few studies have utilized these kinematic parameters in WMH populations, they are considered as sensitive markers for distinguishing gait disorders (25, 26). Our findings extend this evidence to CSVD, highlighting the potential of wearable-based kinematic assessment for early gait impairment detection.

### 4.3 Gait variability and asymmetry

To further explore gait control mechanisms, we quantified gait symmetry and variability using the asymmetry index (AI) and coefficient of variation (CV). We observed a significantly higher asymmetry index of the foot strike angle in patients with moderate-to-severe WMH compared to those with mild WMH, suggesting that WMH severity affects the body’s motor control ability and alters the mechanical properties and movement patterns in both lower limbs. Additionally, CV values for gait speed, stride length, double support, single limb support, swing phase, foot strike angle and toe-off angle were notably higher in patients with moderate-to-severe WMH than in those with mild WMH. This indicates that WMH-related gait abnormalities are linked to gait variability, with worsening WMH severity correlating with greater variability, increased inconsistency of gait parameters, and reduced stability during walking. Previous studies showed that patients with CSVD may experience multidimensional gait disorders, including gait asymmetry and variability to bilateral gait coordination loss (27, 28). This also implies that patients may face a higher risk of falls due to an increase in gait parameters such as the CV and AI.

### 4.4 Trunk and upper limb parameters

Trunk movement and upper limb motion parameters analyzed in this study also suggest that the moderate-to-severe WMH group exhibited increased lumbar spine range of motion during walking, alongside decreased upper limb swing velocity and range of motion. However, these differences between groups were not statistically significant. The difference between our findings and those of previous studies (5, 29) may be attributed to differences in study populations and research methodologies.

### 4.5 Independent predictors of WMH severity

Logistic regression analysis identified toe-off angle, swing phase CV, and age as important markers for distinguishing mild from moderate-to-severe WMH. The emergence of swing phase CV as an independent predictor is noteworthy, as swing phase variability has been shown to be a more effective measure of gait and postural stability than temporal parameters alone (30). The association between increased swing phase CV and WMH severity may be attributed to widespread damage to neural networks in white matter regions, leading to impaired postural control and sensorimotor integration (31, 32). These neural disruptions ultimately manifest as gait disturbances and increased fall risk in affected patients.

### 4.6 Limitations and future directions

Several limitations of this study should be acknowledged. First, the cross-sectional design precludes causal inferences regarding the relationship between WMH progression and gait deterioration. Second, the single-center nature of the study and the male-predominant cohort may limit the generalizability of our findings. Third, although we excluded patients with overt musculoskeletal disorders, subclinical joint conditions may have influenced gait performance. Future multicenter prospective studies with more balanced sex representation and longitudinal follow-up are warranted to validate our findings and to investigate the dynamic relationship between WMH progression and gait changes over time.

## 5 Conclusion

In conclusion, WMH severity significantly influences gait in patients with CSVD, affecting spatiotemporal parameters, kinematic variables, symmetry, and variability. As WMH severity worsens, patients exhibit decreased toe-off angle and increased swing phase CV, both of which are associated with higher fall risk. The anatomical and biomechanical evidence suggests that WMH-related damage to frontal white matter tracts, corticospinal pathways, and sensorimotor integration circuits underlies these gait alterations. Wearable gait analysis systems offer a valuable tool for quantitatively assessing these gait changes and may facilitate early identification of gait impairment in CSVD patients. Future research should focus on interventions targeting these specific gait abnormalities and on evaluating their effectiveness in reducing fall risk.

## Data Availability

The datasets presented in this study can be found in online repositories. The names of the repository/repositories and accession number(s) can be found in the article/supplementary material.
